# Automating data extraction in meta-research: A multi-model benchmark in network psychometrics papers

**DOI:** 10.3758/s13428-026-03052-7

**Published:** 2026-05-22

**Authors:** Benjamin Simsa, Artem Buts, Ivan Ropovik, Matúš Adamkovič

**Affiliations:** 1 Institute of Social Sciences of the Centre of Social and Psychological Sciences, Slovak Academy of Sciences, Košice, Slovakia; 2 Department of Psychology, Faculty of Arts, P. J. Šafárik University, Košice, Slovakia; 3 Faculty of Education, Charles University, Prague, Czech Republic; 4 Institute of Psychology, Czech Academy of Sciences, Prague, Czech Republic; 5 Faculty of Humanities and Social Sciences, University of Jyväskylä, Jyväskylä, Finland

**Keywords:** Large language models, Metaresearch, Network psychometrics, Metascience

## Abstract

Manual data extraction in meta-research is often tedious, time-consuming, and error-prone. In this paper, we investigate whether the current generation of large language models (LLMs) can be used to extract accurate information from scientific papers. Across the meta-research literature, these tasks usually range from extracting verbatim information (e.g., the number of participants in a study, effect sizes, or whether a study is preregistered) to making subjective inferences. Using a publicly available dataset containing a wide range of metascientific variables from 43 network psychometrics papers, we tested five LLMs (Claude 4.6 Opus, Claude 4.5 Sonnet, Claude 4.5 Haiku, GPT-5.2, and GPT-5 mini). We used an automated API-based pipeline to extract variables from the documents. This approach allows batch processing of research papers. As such, it represents a more efficient and scalable way to extract metascientific data than the default chat interface. The extraction accuracy ranged from 79.6% to 91.3% across the models. The extraction performance was generally higher for more explicit, verbatim information and worse for variables that required more complicated inference. Furthermore, most models were able to convey uncertainty in more contentious cases. We provide a comparison of the accuracy and cost-effectiveness of the individual models and discuss the characteristics of variables that are and are not suitable for automatic coding. Furthermore, we describe some of the common pitfalls and best practices of automated LLM data extraction. The proposed procedure can substantially reduce the time and costs associated with conducting meta-research.

## Introduction

Scientific literature continues its exponential growth trajectory, with global output exceeding 3 million articles annually and doubling approximately every 10–15 years (Bornmann & Mutz, 2015; National Science Board, 2022). This proliferation creates an increasingly acute synthesis gap between primary research production and the capacity for comprehensive evidence synthesis (Westgate et al., 2018). Evidence synthesis—whether in the form of systematic reviews, which appraise and synthesize empirical evidence on specific research questions (Higgins et al., 2019), or meta-research, which evaluates the processes, integrity, and outcomes of scientific inquiry itself (Ioannidis, 2018)—is crucial for advancing scientific knowledge and practice. At the core of both endeavors lies data extraction: the systematic identification and coding of relevant information from primary studies into structured datasets. Although both systematic reviews and meta-research depend on accurate data extraction as their shared operational bottleneck, meta-research poses a broader challenge: its variables span verbatim numerical values, binary classifications, and frequently more complex methodological judgments, whereas systematic review extraction is typically governed by more standardized frameworks such as PICO (Patient/Population/Problem, Intervention, Comparison, Outcome; Higgins et al., 2019). This heterogeneity makes meta-research extraction a particularly informative testbed for benchmarking automated tools.

However, metascientific data extraction remains predominantly manual, labor-intensive, and time-consuming. It often takes a year or more and demands significant financial resources per review (Borah et al., 2017). Furthermore, it is susceptible to human error, fatigue, cognitive biases, or subjectivity. This leads to inter-rater variability even under optimal conditions with detailed protocols and training (Belur et al., 2021). Reproducibility studies have documented extraction errors in about two thirds of published pairwise meta-analyses, with such errors altering the statistical significance of pooled results in 7% of cases (Xu et al., 2022). Because of the disparity between the rate of primary research publication and the capacity for manual synthesis creating an ever-widening synthesis gap, valuable knowledge may remain siloed, underutilized, or its integration into the broader scientific discourse significantly delayed (Westgate et al., 2018).

Large language models (LLMs) represent a rapidly advancing class of artificial intelligence that holds considerable promise for addressing these scalability challenges. At their core, LLMs are artificial neural network models pretrained on vast text corpora to capture linguistic patterns and factual knowledge, mapping language into dense vector spaces where similarity reflects meaning (Mikolov et al., 2013), and leveraging attention mechanisms to weigh the importance of different parts of input text. Contemporary decoder LLMs demonstrate sophisticated abilities including advanced natural language understanding for interpreting complex queries and text, natural language generation for producing coherent and contextually relevant outputs, contextual reasoning for drawing inferences, and information extraction for identifying and isolating specific pieces of data from text.

A rapidly growing body of empirical work has begun to map the capabilities and limitations of LLMs across diverse research tasks. In text annotation, zero-shot LLM outperformed crowd-sourced human annotators by an average of 25 percentage points across classification tasks at roughly one-thirtieth of the cost (Gilardi et al., 2023). LLMs have also shown promise for qualitative deductive coding, achieving agreement levels comparable to human coders (Chew et al., 2023), with codebook-centered prompting designs outperforming example-based approaches (Xiao et al., 2023). LLMs have further demonstrated strong performance in sentiment analysis, where ensemble approaches combining them with transformer models achieved over 86% accuracy on cross-lingual data (Miah et al., 2024). Performance on inductive coding, however, was weaker, with models producing 22–39% duplicative or superficial codes (Parfenova et al., 2025). LLMs have also been applied across the evidence synthesis pipeline. A scoping review found that 41% of studies used LLMs for literature search, 38% for screening, and 30% for data extraction, with GPT architectures dominating 89% of evaluated studies (Lieberum et al., 2025). For screening, LLMs reduced manual workload by 33–93% depending on prompt specificity (Delgado-Chaves et al., 2025). For data extraction, even Claude 2 achieved 96.3% accuracy across 160 clinical trial data elements, with high test–retest reliability (Gartlehner et al., 2024), and a subsequent prospective study demonstrated artificial intelligence (AI)-assisted extraction to be non-inferior to human-only extraction while being faster and less costly (Gartlehner et al., 2025). Collectively, this literature indicates that while LLMs can achieve and sometimes exceed human-level performance on structured research tasks, their reliability degrades on tasks requiring interpretive judgment, and it may take some time before they are ready for fully autonomous deployment in formal research protocols (Lieberum et al., 2025).

LLMs may be particularly useful for meta-research, which frequently involves labor-intensive tasks. For example, meta-research frequently entails large-scale screening of scientific literature, including the extraction and coding of diverse information about how primary studies are designed, conducted, analyzed, and disseminated (Hardwicke et al., 2020; Ioannidis, 2018). More specifically, LLMs may assist in identifying and systematically coding data related to study methodology (e.g., specifics of sampling, randomization and blinding methods, procedure details, missing data treatment), details of outcome definition and measurement (e.g., primary and secondary outcomes, various psychometric properties of measures employed), the thoroughness and transparency of reporting (e.g., adherence to established reporting guidelines, declarations of conflicts of interest, ethical approval, sources of funding, concordance with preregistered protocols), the specifics of how statistical results are presented (e.g., use of analytic methods, reporting of effect sizes and inferential statistics), or the adoption of open science practices (e.g., preregistration of studies, availability and accessibility of research data, code, and materials), among other elements vital for a comprehensive assessment of research practices. The capacity to extract such detailed information at scale is fundamental to achieving core meta-research objectives, such as evaluating methodological quality across fields, assessing research biases, and investigating factors related to research credibility and reproducibility (Hardwicke et al., 2020; Ioannidis, 2018). Taken together, these capabilities illustrate how LLMs can be integrated into the meta-research process, primarily by assisting in the systematic collection, coding, and structuring of data about research itself at scale (Scherbakov et al., 2025).

The allure of enhancing the speed, scalability, and consistency of metascientific research is tempered by concerns regarding the operational reliability and validity of LLMs (Ashwin et al., 2023; Roberts et al., 2024; Tang et al., 2024). These models are known for their propensity to “hallucinate,” confidently fabricating details not present in the source text, and to exhibit biases ingrained from their training data, potentially perpetuating or amplifying existing societal or methodological biases (Ji et al., 2023; Roberts et al., 2024). Performance may also be markedly influenced by the intricacies of prompting, where subtle variations in wording, the inclusion and quality of examples (few-shot learning), and output format specifications can lead to markedly different outcomes (Valdenegro, 2023). This is compounded by “prompt drift,” where a previously optimized prompt degrades in performance as the underlying model is updated—a documented phenomenon that poses significant challenges for reproducibility (Chen et al., 2023). Further issues include the lack of interpretability inherent in their “black box” nature, which hinders error diagnosis (Luo & Specia, 2024), difficulties in handling (poorly formatted) complex document structures like tables, and the risk of overreliance on unverified outputs (Carnat, 2024). Importantly, these limitations should be evaluated against the human alternative. Manual data extraction is also imperfect, susceptible to error, fatigue, and cognitive biases, which can lead to significant inter-rater variability (Belur et al., 2021). Therefore, the central issue is how LLMs’ performance and specific error profile compare to those of human coders. Consequently, understanding the accuracy of LLMs is the first step in determining how they can be most effectively and responsibly integrated into research workflows.

Despite the rapid proliferation of LLMs, systematic empirical investigations into their accuracy for the specific data extraction tasks common in meta-research remain scarce. Recent scoping and systematic reviews confirm that while research on LLM applications in evidence synthesis is accelerating, it remains largely in an exploratory phase (Lieberum et al., 2025; Scherbakov et al., 2025). While researchers are beginning to explore the use of LLMs for various evidence synthesis tasks across multiple disciplines, this is primarily focused on discrete steps of the systematic review process (O’Connor et al., 2024). In contrast, comprehensive benchmarks that systematically compare multiple state-of-the-art LLMs on a standardized set of variables pertinent to the broader field of quantitative meta-research are still lacking. This gap in the literature hinders the development of evidence-based guidelines for an effective implementation of LLMs in the domain of metascience. Without such research, the risk of misapplication, overreliance on unverified outputs, or the introduction of systematic errors into the evidence synthesis pipeline remains substantial.

## The present study

This study aimed to address this critical gap by systematically evaluating the accuracy of a range of contemporary LLMs in extracting diverse quantitative and methodological variables from a corpus of published psychological research papers. The primary objective was to quantify the performance of these models against a human-coded reference dataset, thereby providing robust empirical evidence on their current capabilities and limitations for metascientific data extraction. To do so, we evaluated five contemporary LLMs from two major providers: Anthropic’s Claude 4.6 Opus, Claude 4.5 Sonnet, and Claude 4.5 Haiku, and OpenAI’s GPT-5.2 and GPT-5 mini.

The core of our investigation was a direct comparison of LLM-generated outputs against a human-coded reference dataset, using a meta-research dataset by Blanchard et al. (2022), which reviewed and coded 43 experience sampling method studies. This dataset provides a robust and ecologically valid benchmark, with variables ranging from simple numerical values (e.g., sample size) to more nuanced categorical judgments (e.g., statements on open science practices). Our methodology involved submitting the full-text PDF of each article to each LLM via a single, structured prompt that was developed through an iterative refinement process. The LLMs’ structured outputs were then verified by human evaluators against the reference dataset. The subsequent analysis aimed to quantify overall and variable-specific accuracy, compare performance across different models and variable types, and examine factors influencing LLM reliability.

Another contribution of the present paper is the full automation of the data extraction process. While previous work (Gartlehner et al., 2024) mainly used the chat interface to ask the models questions about the uploaded PDF version of scientific papers, this approach inherently limits the scalability of further LLM-powered meta-research projects. Therefore, we developed a procedure that allows researchers to process an entire set of scientific papers into the resulting dataset using a single Python script. This is achieved through a combination of an API-based approach and a prompt structured to force the output format of each API output to conform to JSON format. The JSONs (containing all extracted variables for the given paper) are then concatenated into a single dataset.

## Methods

### Sample

We used the existing human-coded dataset coded by Blanchard et al. (2022) as the reference for evaluation of the LLM-extracted metascientific data. We did not conduct a full new manual coding of the papers for the benchmark. We selected this dataset as it contains a diverse set of variables representative of meta-research in psychology: binary variables (e.g., yes/no answers about whether a certain open science practice was used), categorical data (e.g., types of samples), and free-form text (e.g., sample sizes used for the network estimation).

The Blanchard et al. (2022) dataset is derived from a systematic scoping review of 43 psychological studies published before April 2021 that used the network psychometrics approach on intensive longitudinal data (i.e., psychological data collected via the experience sampling method [ESM]/ecological momentary assessment [EMA]). Blanchard et al. aimed to systematically assess the research, analytical, and reporting practices used in this literature. All papers included in the review use broadly similar statistical approaches to analyze similarly structured intensive longitudinal data, usually within the field of clinical/health psychology. However, they also differ in important aspects: sample characteristics, data collection schedules, the substantive questions, and reporting practices.

We did not include the full set of variables used by Blanchard et al., as this study was designed as a benchmark of LLM-based extraction, rather than a reproduction of the full coding scheme used by Blanchard et al. (2022). The included variables were selected to reflect the diverse range of variable types encountered in metascientific data, while remaining feasible for manual verification and comparative grading across models.

In the present study, the extracted metascientific data provided by Blanchard et al. were used as a reference standard for quantitative evaluation of the LLM responses. We do not treat this dataset as an error-free ground truth, but as an operational benchmark derived from prior human coding.

### Evaluated models

We evaluated the following LLMs in this study:

- Anthropic Claude 4.6 Opus
- Anthropic Claude 4.5 Sonnet (version claude-sonnet-4–5–20250929)
- Anthropic Claude 4.5 Haiku (version claude-haiku-4–5–20251001)
- OpenAI GPT-5.2 (version gpt-5.2–2025-12–11)
- OpenAI GPT-5 mini (version gpt-5-mini-2025–08-07)

The models were chosen to represent the selection of then-current options from the two major providers—OpenAI and Anthropic. We aimed to include both the large flagship models (Opus, GPT-5.2) and models representing different tiers in each AI lab’s lineup. For the three Claude models, we set the temperature to 0.0 in order to maximize the reproducibility of the results. Setting the temperature was not available for the two OpenAI models.

### Prompting

We designed a single structured prompt to extract data for all predefined variables from each article (i.e., all the relevant variables were extracted from *one* article per one API call). We developed the prompt through an iterative process of both consulting with domain experts in psychology and metascience and testing the performance of the prompt manually on a subset of five randomly selected articles from the dataset. Prompt development was also informed by official OpenAI prompt guidance available at the time of study design (OpenAI, n.d.). In line with this guidance, we structured the prompt to place task instructions up front, specify the contextual scope of extraction, define the expected JSON output format, and explicitly instruct the model to return “check” rather than guess when the source text did not support a confident answer or the model could not find one. The full prompt is included in the supplementary materials (https://osf.io/eqt78/).

When formulating the definitions of the specific variables to be extracted, we aimed to stay as close as possible to the coder manual used by Blanchard et al., (2022; available at https://osf.io/mpj89). However, for most extracted variables, it was necessary to provide further context to the LLM. For example, while Blanchard et al. (2022) explicitly focused on assessing methods used for collection and analysis of ESM data, some of the analyzed articles contained multiple datasets collected by different methods (e.g., neuroimaging approaches or cross-sectional self-report questionnaires). As a result, we had to specify that we were interested solely in ESM data in the prompt. For example, for item 22 (collection device), the phrasing of the question in the Blanchard et al. (2022) coder questionnaire was “What device was used to collect data?” (Blanchard et al., 2022), while the version we used for the LLM extraction was as follows:

*Variable name: 22_collection_device*

*Response format: string*

*Question: What type of device was used to collect the (intensive longitudinal/ESM/EMA/ambulatory) data used in the network analysis? Please select from the following categories: “Smartphone”, “Palmtop”, “Digital watch”, “Paper and pen”, “Computer”, “Psymate”, “iPod”. Please note that multiple device types might have been used in the analyses. If that is the case, please list all of them.*

We also tuned the prompt to avoid unwanted behaviors by the models encountered during the prompt piloting phase. For the preregistration variable, the variable “preregistration” was only stated as “*75. Preregistration?*” in the coding questionnaire used by Blanchard et al. (2022), but we used the following formulation in the prompt:

*Variable name: 75_preregistered_yn*

*Response format: 1 (yes)/0 (no)*

*Question: Was the present scientific paper registered, pre-registered or post-registered? That is, is a registration available online, for example on Open Science Framework - OSF, or AsPredicted) according to the information included in the paper? Information about the study being preregistered is often (but not always) located near to a link to websites such as the Open Science Framework (osf.io) or AsPredicted. Please note that by “registration” we only mean a registration of hypotheses and methods before collecting or analyzing the data. Please do not confuse this with a general registration of a clinical trial - they are not the same thing.*

In addition to the variable definitions, we explicitly assigned the role of a “meticulous research assistant” tasked with metascientific data extraction in psychology. We framed the task as extraction from a set of psychological studies that use intensive longitudinal data for network analyses. Importantly, we instructed the model to only extract the data based on information explicitly stated in the paper, output “check” whenever uncertain (rather than guess the datapoint), and provide the verbatim text from which the extracted datapoint was derived.

The prompt was developed specifically for the use case of this paper, i.e., extracting a specific set of metascientific variables from scientific papers within a relatively narrow scientific field. However, we have followed a general logic that can be extended to other extraction tasks. As a general principle, the process of prompt-writing and adaptation should start with formulating a clear operational definition for each target variable. To ensure adherence to the desired output format, it is important to specify the expected response format and coding conventions, as well as rules for dealing with missing or ambiguous information. From our experience, it is crucial to explicitly allow the model to express uncertainty in the responses/abstain from responding when not certain enough, as the models tend to hallucinate the response in the absence of relevant information instead. Another helpful step is requiring an “evidence” column for each extracted variable. The model should provide verbatim information from which its extracted response was derived. This makes the inspection and grading of the LLM output much more efficient and transparent and can aid in understanding the common error patterns.

### Data extraction and grading

The LLMs extracted the metascientific variables from the 43 scientific articles based on a structured prompt. We, submitted the original PDF full-text versions of the articles to each model via API file input. The models were instructed to return their outputs in JSON format. For each extracted variable, the models were also asked to provide a self-generated confidence score on a scale of 0 to 1, representing their certainty in the accuracy of the extracted information. The individual JSON outputs, one for each model–article pair, were subsequently parsed and aggregated into a single comprehensive tabular dataset. This consolidated format facilitated straightforward comparative analysis across models and variables. This programmatic API-driven approach offers significant advantages over manual extraction via chat interfaces—primarily in terms of scalability for large numbers of documents and reproducibility of the extraction process.

The accuracy of the information extracted by each LLM was subsequently manually verified against a reference standard derived from prior human coding. A standardized coding scheme was applied to each extracted datapoint:

- 1 (Correct): The extracted information was deemed fully accurate and complete when compared to the manually coded reference data.
- 0 (Incorrect): The extracted information was inaccurate, partially true but critically incomplete, or contained additional incorrect details.
- NA (Check): The model explicitly indicated uncertainty in its response (e.g., by outputting the string “check”) or provided a response that could not be coded in a binary fashion regarding its correctness—in a real-life metascientific data extraction scenario, any paper–variable combination for which the model output “check” would be manually assessed by a human coder.

We only considered the response as correct when it was fully accurate. For example, for variables where a list with multiple elements had to be extracted (e.g., the software packages used for the network estimation), we only graded the response as 1 when the reference data and the model response fully matched. When the model response included extra information and/or missed any elements contained in the reference data, we marked the model response as incorrect. We considered semantic accuracy during the grading process—for example, for the variable *25_n_timepoints*, when the reference data was “2” (i.e., the ESM measurement was taken twice per day), we considered responses such as “2 times a day” or “twice daily” as correct. The assigned grades for each model response, as well as the per-variable criteria used for grading, are available in the OSF repository associated with this paper.

To refine the reference datapoints in the reference dataset, we conducted a manual verification step. After we carried out the initial grading of the LLM-extracted data, we aggregated the results on the paper × variable level and flagged the cells where the majority of models were incorrect in their responses. We then manually rechecked the flagged cells against the full-text version of the given source paper and the reference dataset from Blanchard et al. (2022). For each cell, we recorded one manual verification decision: *GT_OK* (retain the datapoint from the reference dataset), *GT_FIX* (revise the reference dataset), *GRADE_FIX* (retain the reference datapoint but correct grading logic), or *GRADE_FIX_NA* (retain the reference datapoint and mark grading as non-scorable). Then we saved the revised reference datapoints to an updated reference table and regraded the LLM responses associated with the revised reference dataset cells. There were 65 cells included in the manual verification procedure (across 35 papers and 16 variables). For 64.6% of these, the original reference datapoint was deemed valid, and for 30.8%, we decided to modify the corresponding cell in the reference dataset. The remaining cases were assigned a *grade_fix* decision. The manual verification aimed to reduce the possibility that apparently incorrect model data extractions reflected problems in the original reference dataset instead of an error on the model side.

### Open science practices

The full prompt and the Python code used for the data extraction, as well as the resulting data and the R script used for the preprocessing, analysis, and visualization of the results, are available at https://osf.io/eqt78/.

## Results

We present the results in three parts: overall and variable-specific extraction accuracy, multilevel modeling of performance predictors, and confidence calibration analysis.

### Overall model performance

The overall performance of the LLMs in accurately extracting information across all included variables, ordered by the proportion of correct responses, is reported in Table 1. Two Anthropic models (Claude 4.6 Opus and Claude 4.5 Haiku) emerged as the two models with the greatest accuracy (91.3% correct responses for Opus, 90.6% for Haiku). Below, we describe the data extraction accuracy for the different variables and variable × model combinations.

**Table 1:** The overall performance of the LLMs in accurately extracting information across all included variables, ordered by the proportion of correct responses

Model	Correct	False	Check	No. of valid responses
Claude 4.6 Opus	91.3%	7.3%	1.4%	772
Claude 4.5 Sonnet	88.9%	10.1%	1.0%	773
Claude 4.5 Haiku	90.6%	8.7%	0.6%	770
GPT-5.2	88.7%	8.8%	2.5%	772
GPT-5 mini	79.6%	9.3%	11.0%	771

“No. of valid responses” refers to the number of model–paper–variable combinations included in the grading procedure. These responses were coded as Correct, False, or Check. Missing outputs, i.e., cases in which the model did not provide any output for a given variable, were excluded

### Performance by variable

Extraction accuracy varied substantially depending on the specific variable (see Table 2). For example, the title of the paper was extracted with 100% accuracy across all models. Other variables demonstrating high average correctness included the sample size of the ESM sample (98.1%) and the number of timepoints per day (97.2%), i.e., the variables that were usually described very explicitly in the papers. Conversely, variables such as the packages used for the estimation (72.6% correct), whether the analytical code was declared to be publicly available (75.4% correct, but with a high 16.6% “check” rate), and the devices used for collecting ESM data (75.5% correct) were found to be more challenging for the models.

**Table 2:** Extraction accuracy per extracted variable (across all models)

Variable	Variable description	Correct	False	Check	No. of valid responses
Title	Full paper title	100.0%	0.0%	0.0%	215
Sample size	Sample size used for network estimation	98.1%	1.9%	0.0%	215
Timepoints per day	Number of ESM measurement occasions per day	97.2%	2.8%	0.0%	215
Visualization packages	Software packages used for network visualization	96.7%	3.3%	0.0%	215
Number of nodes	Number of nodes in the estimated networks	96.3%	3.7%	0.0%	215
Number of days	Number of ESM collection days	94.0%	2.8%	3.3%	215
Network types	Which network types were estimated (contemporaneous, temporal, between-person)	94.0%	6.0%	0.0%	215
DOI	Digital object identifier of the paper	93.0%	6.5%	0.5%	215
Software	Software used for data analysis	92.9%	7.1%	0.0%	211
Sample type	Whether the sample was clinical/health-related or nonclinical	90.7%	9.3%	0.0%	214
Centrality test	Whether a (formal statistical) test of centrality differences was used	87.0%	12.1%	0.9%	215
Data declared available	Whether the paper declared the data to be publicly available	84.7%	7.9%	7.4%	215
Preregistered	Whether the paper reported a study registration (pre-/post-registration)	83.7%	6.5%	9.8%	215
Redundancy reported	Whether the authors checked for redundancy or conceptual overlap between nodes	76.7%	13.5%	9.8%	215
Collection device	Type of device used to collect the ESM/EMA data	75.5%	20.3%	4.2%	212
Code declared available	Whether the paper declared the analysis code to be publicly available	75.4%	8.1%	16.6%	211
Country	Country or countries where the data collection took place	72.6%	20.0%	7.4%	215
Estimation packages	Software packages used for network estimation	72.6%	27.4%	0.0%	215

These between-variable differences provide context for the interpretation of the overall performance of the models. The relatively straightforward variables (e.g., paper title, sample size) were extracted at near-ceiling levels. On the other hand, the more nuanced variables that often required more than just the extraction of verbatim information (e.g., collection devices or estimation packages) were considerably more difficult for the models to extract. Therefore, the overall model accuracy reflects the strong performance on the explicit/easy-to-extract variables and should not be interpreted as evidence for equally high performance across all extracted variables.

### Performance per model–variable combination

**Fig. 1:**
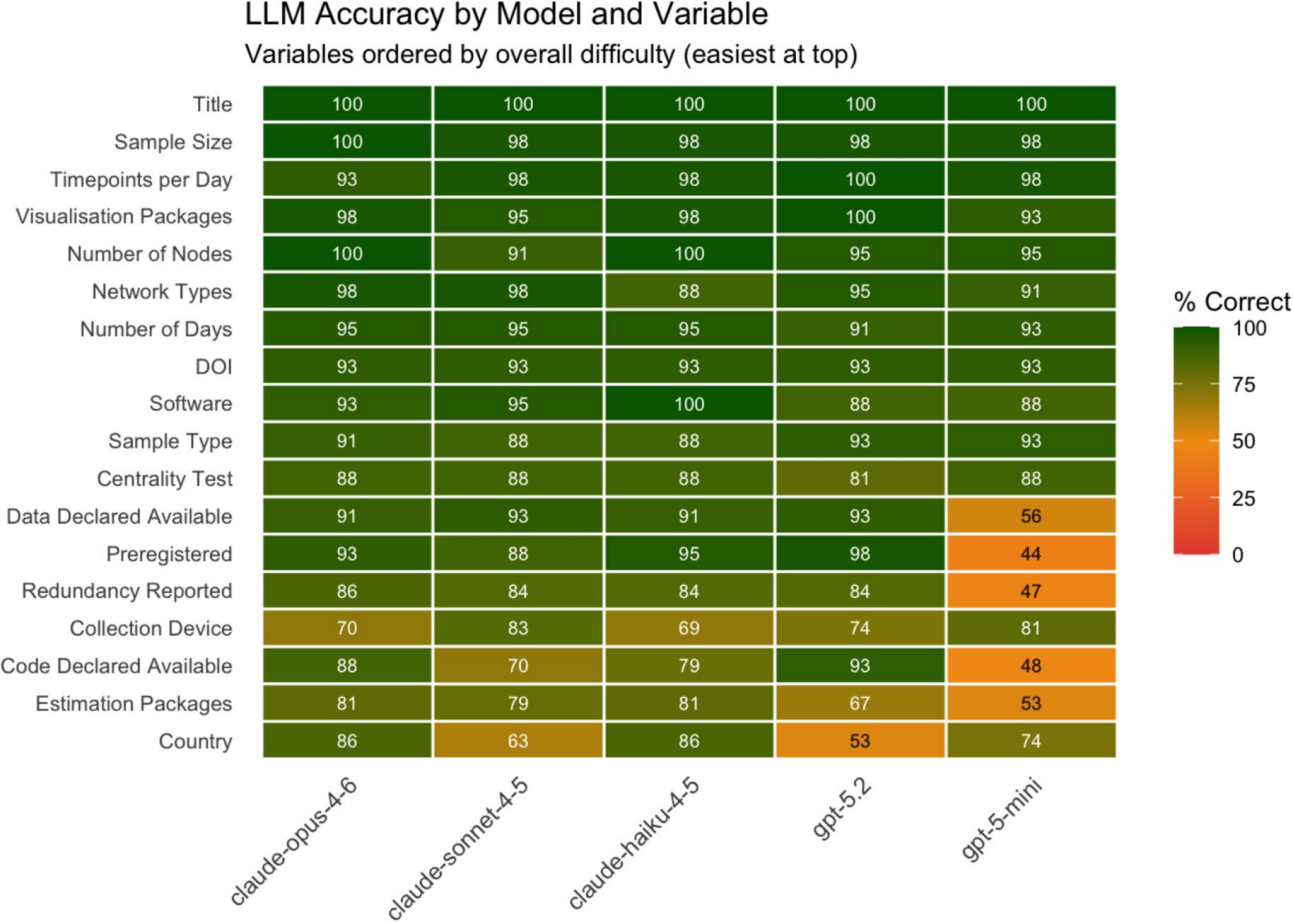
Heatmap illustrating the accuracy of each LLM for each specific variable, with variables ordered by overall difficulty

Figure 1 presents a heatmap illustrating the accuracy of each LLM for each specific variable, with variables ordered by overall difficulty. This visualization revealed nuanced performance patterns; for instance, GPT-5.2, despite ranking fourth in overall accuracy, outperformed the top two Claude models on the binary variables pertaining to publicly available preregistration, code, and data.

### Multilevel modeling results

To obtain a more generalizable estimate of model performance while accounting for the inherent variability introduced by different scientific papers (file) and the specific information being extracted (variable), we employed a generalized linear mixed model (GLMM). We used the model as a predictor, allowing for random intercepts for the different papers (files) and variables. This approach allowed us to quantify the unique contribution of each LLM to extraction accuracy, statistically controlling for the nested structure of the data (i.e., multiple observations per paper and per variable type). The model predicted the log-odds of a correct extraction (correct = 1, incorrect = 0), with GPT-5 mini serving as the reference category for model comparisons.

The GLMM analysis confirmed significant performance differences among the LLMs. The reference model was GPT-5-mini, the worst-performing model. Compared to the reference, odds ratios were highest for Claude 4.6 Opus (OR = 3.10), followed by Claude 4.5 Haiku (OR = 2.83), Claude 4.5 Sonnet (OR = 2.29), and GPT-5.2 (OR = 2.24). The reference intercept indicates relatively high baseline accuracy for GPT-5 mini (log-odds = 1.764; *p* <.001). We report the full fixed-effect results in Table 3.

**Table 3:** Multilevel modeling results (fixed effects for the different LLMs)

Predictor	Estimate (log-odds)	*SE*	*z*	95% CI—lower	95% CI—upper	OR
Intercept (GPT-5 mini baseline)	1.764	0.305	5.775	1.165	2.362	5.833
Claude 4.5 Haiku	1.043	0.161	6.464	0.727	1.360	2.838
Claude 4.6 Opus	1.134	0.165	6.891	0.811	1.456	3.107
Claude 4.5 Sonnet	0.832	0.154	5.391	0.530	1.135	2.298
GPT-5.2	0.809	0.154	5.262	0.508	1.111	2.246

The rank order of performance based on mixed-effects model estimates was as follows: Claude 4.6 Opus > Claude 4.5 Haiku > Claude 4.5 Sonnet > GPT-5.2 > GPT-5 mini. However, each model–paper pair was evaluated only once. Therefore, this ordering should be interpreted with caution, especially for models with similar performance.

The random-intercept variance was substantially larger for variable than for paper, indicating that differences in extraction difficulty across variables contributed more to performance variation than differences across papers.

#### Cost-effectiveness

We provide the total estimated cost as of the time of data extraction in Table 5. Claude 4.6 Opus was the most expensive ($10.70 total cost), followed by Claude 4.5 Sonnet ($6.66). These two Anthropic models accounted for around 77% of the total price spend. Mid-cost models were GPT-5.2 ($2.41) and Claude 4.5 Haiku ($2.18). GPT-5 mini was by far the most cost-minimizing model, with total spend for extracting data from the 43 papers being $0.71 for this model. These estimates use standard API rates valid as of March 6, 2026, without caching or batch discounts.

### Confidence calibration

Descriptively, the average confidence scores were high for all models (see Table 4). The average confidence reported by Claude 4.6 Opus (as the model with the most accurate responses) was 0.921, while its success rate was 0.926.^1^ Claude 4.5 Sonnet assigned the highest confidence to its responses (0.964, success rate 0.898), while GPT-5.2 was the least confident model (0.911 overall confidence, 0.910 success rate). GPT-5 mini was the most overconfident model, reporting high overall confidence (0.956) despite having the worst success rate (0.895).

**Table 4:** Brier scores for confidence calibration and the proportion of missing confidence scores

Model	Mean confidence	Brier score	AUC (resolution)	No. calibration	Missing confidence scores (where non-missing response)
Claude 4.6 Opus	0.921	0.064	0.767	761	0.0%
Claude 4.5 Sonnet	0.964	0.093	0.620	765	0.0%
Claude 4.5 Haiku	0.920	0.086	0.673	765	0.0%
GPT-5.2	0.911	0.086	0.630	753	0.0%
GPT-5 mini	0.956	0.112	0.561	686	0.0%

#### Brier scores, calibration curves, and AUC (resolution)

To evaluate the models’ ability to reliably provide confidence scores for their answers, we carried out a calibration analysis using two methods: plotting calibration curves and comparing Brier scores (Brier, 1950). The Brier score is computed as the mean squared difference between the assigned confidence (i.e., the probability of the answer being correct) and the actual outcome. As such, a low Brier score indicates that a model is better able to accurately estimate the probability of its answer being correct.

Claude 4.6 Opus and Claude 4.5 Haiku emerged as the two models with the lowest Brier scores (0.0639 and 0.0859, respectively), with GPT-5.2 essentially identical to Haiku (0.0860). GPT-5 mini showed the poorest calibration (0.1120), with Claude 4.5 Sonnet second to last (0.0927). The ranking based on Brier scores is mostly in line with the overall accuracy ranking shown above.

For the calibration curves, we divided the confidence scores into 10 bins (0–0.1, …, 0.9–1.0). We then computed the proportion of correct answers for each of the bins. The curves show the mean model-assigned confidence within the given bin (*x*-axis) against the observed proportion of correct answers (*y*-axis). Points located below the diagonal dashed line indicate model overconfidence (i.e., the assigned confidence is higher than the observed one).

Furthermore, to distinguish between calibration and resolution, we computed the area under the receiver operating characteristic curve (AUC) as a metric of resolution. In this context, AUC measures how well a confidence score provided by a model discriminates between correct and incorrect data extractions (independently of absolute calibration).

Overall, the models tended toward mild overconfidence. Most responses were assigned high confidence (especially so in the high-confidence bins), but model behavior differed. Claude 4.5 Sonnet and GPT-5 mini showed the clearest overconfidence overall, whereas Claude 4.6 Opus was slightly underconfident on average. GPT-5.2 appeared near-calibrated in aggregate, but this reflected offsetting bin-level errors: it was underconfident in lower- to mid-confidence bins and overconfident in the highest-confidence bin. Confidence-score compliance was complete in the final dataset, with 0% missing confidence values across all models (Table 4).

**Fig. 2:**
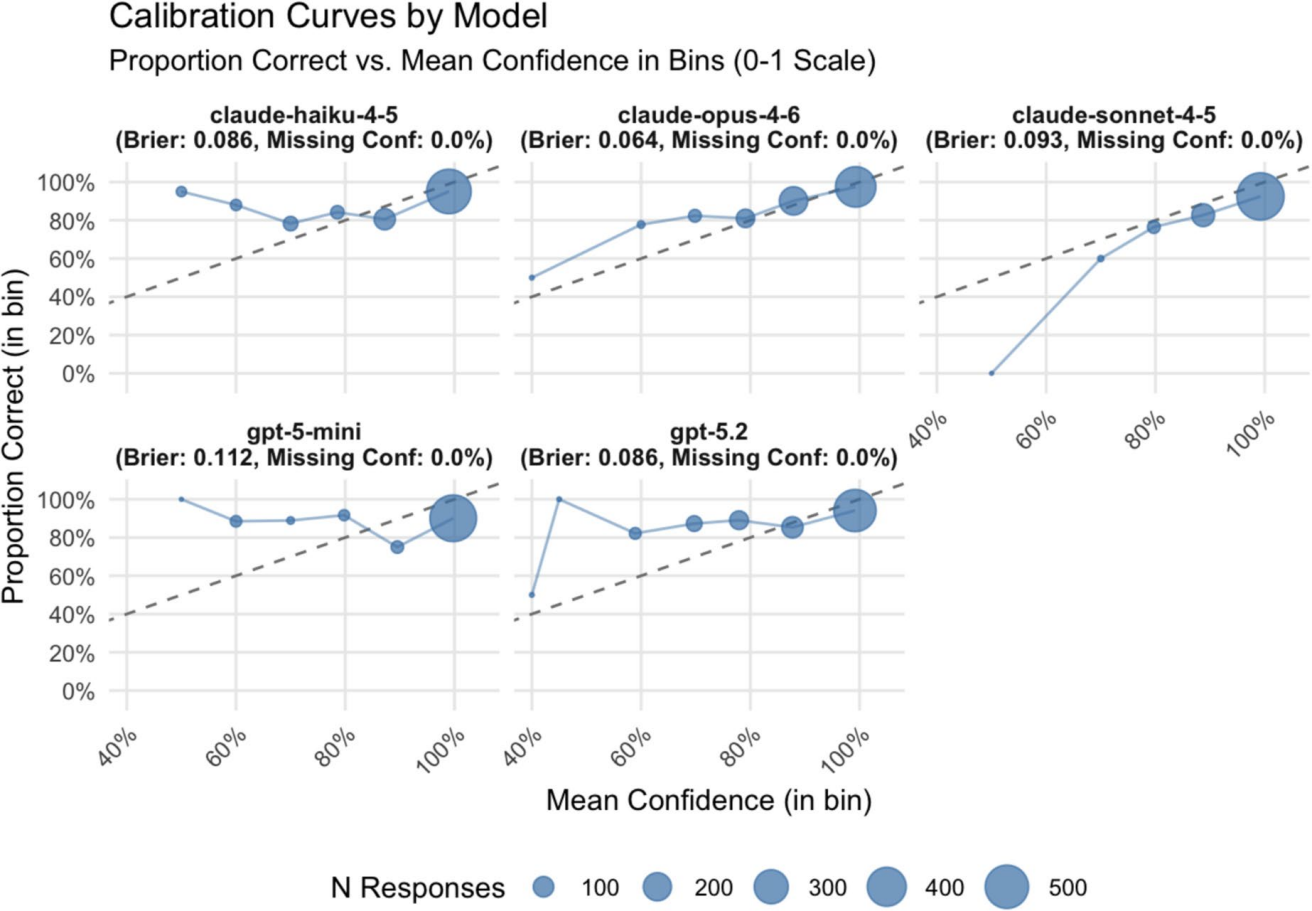
Calibration curves for the large languages models

The three methods for analyzing confidence led to a similar overall ordering of models. Claude 4.6 Opus showed both the lowest Brier score (0.0639) and the highest AUC (0.767), which indicates the strongest confidence performance. It was followed by Claude 4.5 Haiku (Brier = 0.0859, AUC = 0.673), GPT-5.2 (Brier = 0.0860, AUC = 0.630), and Claude 4.5 Sonnet (Brier = 0.0927, AUC = 0.620). GPT-5 mini performed worst on both indices, providing confidence ratings with very limited information about whether an extraction was really correct (Fig. 2).

## Discussion

This study systematically evaluated the performance of five leading LLMs on a real-world metascientific data extraction task. In this benchmark, conducted in a subfield of psychological research, the best-performing models achieved relatively high accuracy for one-shot extraction of a range of metascientific variables from full-text papers (degrading slightly when complex interpretation or synthesis of scattered information was needed). Importantly, this was done with a fraction of the time and cost typically required for manual coding in meta-research. However, the performance was highly heterogeneous between variables. The models provided the most accurate extractions for information explicitly stated in the full-text versions of the papers, and lower accuracy for variables that required more interpretation and judgment (Table 5).

**Table 5:** Estimated costs of data extraction (i.e., the total cost of all output and input tokens at the time of extraction)

Model	Input tokens	Output tokens	Total tokens	Input $/1 M	Output $/1 M	Input cost ($)	Output cost ($)	Total cost ($)
Claude 4.6 Opus	1,836,456	60,807	1,897,263	5.00	25.00	9.18	1.52	10.70
Claude 4.5 Sonnet	1,836,327	76,519	1,912,846	3.00	15.00	5.51	1.15	6.66
Claude 4.5 Haiku	1,836,327	69,515	1,905,842	1.00	5.00	1.84	0.35	2.18
GPT-5.2	905,892	59,253	965,145	1.75	14.00	1.59	0.83	2.41
GPT-5 mini	905,892	242,379	1,148,271	0.25	2.00	0.23	0.48	0.71

The per-token costs were valid as of February 2026. The differences in the number of input tokens between the Anthropic and OpenAI models are due to the differences in provider-specific tokenization and token-accounting methods. Costs are standard API estimates (no caching or batch discounts)

Although it is still not yet advisable to fully rely on the results of automated data extraction (since even the best-performing model got 1 in 10 responses wrong), the procedure outlined in this paper represents a streamlined approach to improved metascientific data extraction in similar benchmark settings. Given that median total error rates for human coding are approximately 25% (Mathes et al., 2017), implementing a hybrid pipeline could enhance both the scalability of meta-research workflows and the cross-validation of coding accuracy. For instance, an LLM (or an ensemble of multiple models) can function as a correctness check for human coding—when outputs from multiple state-of-the-art LLMs contradict manually coded data, such discrepancies can serve as a signal to reverify the coding results. In practice, this could involve running two or more models in parallel and flagging any paper × variable cell where models disagree or where confidence falls below a predetermined threshold (e.g., 0.85) for human adjudication. Beyond serving as a verification tool, LLMs can also be used to expand the scope of meta-research projects. Preserving the human-in-the-loop approach, human coders could extract data from a small sample of the literature and then allow LLMs to code a much larger portion of the available papers. The accuracy of LLM coding would then be assessed against the human-coded benchmark (as demonstrated in the present project), and only variables with sufficient accuracy would be used in further analyses.

We also asked the models to assign a confidence score to each extracted datapoint. These results can provide useful information about whether the models themselves can signal the reliability of the extracted data. Recent work suggests that while confidence verbalized by LLMs can carry some information, the confidence ratings are often not aligned with actual correctness and the models often tend toward overconfidence (Huang et al., 2024; Xiong et al., 2024). Our results are in line with this broad pattern. Mean reported confidence was high across all models. While Claude 4.5 Sonnet and GPT-5 mini exhibited the largest gaps between the overall confidence and success rate, Claude 4.6 Opus and GPT-5.2 provided an overall confidence score very similar to their respective success rates. Furthermore, the AUC results show that the better-performing models are more likely to assign a high confidence to correct than to incorrect extracted datapoints. Therefore, reported confidence can provide some signal (e.g., for prioritizing human review of the extracted data), but not as a standalone metric for assigning a degree of trust to the extracted data.

### Limitations

While the present findings offer granular, empirical evidence to support a cautiously optimistic perspective on the use of LLMs in meta-research, several factors point to promising avenues for future exploration. The imperfect accuracy observed in this study (75–90%) likely reflects a combination of interacting factors rather than a single limiting source. These include properties of the variables (e.g., whether information is explicitly stated versus requiring interpretation), characteristics of the source papers, the design and specificity of the prompt, and model-specific limitations in handling ambiguity or integrating information across the document. While our results suggest that variable type plays a major role, the present design does not allow these sources of error to be fully disentangled.

We used a single one-shot prompt to extract all predefined variables from each article because our aim was to benchmark a scalable batch-extraction workflow rather than a multistep agentic or decomposed prompting pipeline. We did not formally compare this design against shorter prompts, explicit role-prompt variants, or single-question-per-request workflows. Accordingly, we cannot determine from the present study whether such alternatives would have improved extraction accuracy or reduced hallucinated outputs in this specific task.

Notably, human coders in this study had the freedom to consult supplementary files, follow citation trails, or access external databases, whereas LLMs were limited to the text of the primary document and whatever background knowledge they retained from pretraining. This asymmetry likely depresses the models’ true potential, suggesting that figures reported here should be interpreted as lower-bound estimates of what the same models could achieve with full retrieval support.

Importantly, the observed differences in performance across variables should not be interpreted as reflecting intrinsic extraction difficulty alone. They may also be influenced by how individual variables were operationalized in the prompt (e.g., the level of contextual detail or specificity provided). As a result, variable-level performance reflects a combination of these factors, which cannot be independently assessed within the present study. Future work could examine whether extraction errors systematically cluster in papers with specific reporting styles or structural characteristics, which could inform targeted prompt refinements or help identify persistent model limitations.

Additionally, all models were prompted using the same one-shot instructions, without tailoring for model-specific optimization strategies or providing in-context examples. Future studies could examine whether prompt engineering aligned with each model’s design, or few-shot prompting with annotated examples, yields substantial improvements in accuracy. A related limitation is that each model–paper pair was evaluated using a single extraction run. As such, the present results reflect one-shot performance under fixed prompting conditions rather than a direct estimate of run-to-run variability in model outputs. This design is informative for an applied use case in which a researcher runs the extraction pipeline once. However, it does not warrant strong conclusions about the stability of small performance differences between closely matched models. Thus, we interpret the observed ordering of top-performing models cautiously and we treat it as benchmark-specific rather than a definitive ranking. Future work could extend this design by repeating extractions under identical conditions to quantify within-model variability more directly.

The present evaluation was confined to a single dataset of 43 papers drawn from a narrow subfield of psychology with specific reporting conventions, terminology, and article structure. Broadening this research across disciplines and document types will be key to assessing the generalizability and boundaries of LLM-assisted meta-research.

Furthermore, our study focused on meta-research rather than systematic review extraction. The core findings—that LLMs excel on explicitly stated variables and struggle with those requiring inference, and that confidence scores can usefully triage uncertain cases—are likely to generalize, since both domains share the same extraction bottleneck. However, systematic reviews place higher stakes on numerical precision (e.g., effect size data for meta-analytic pooling), where even small extraction errors propagate into pooled estimates.

Lastly, we need to note that the abilities of the state-of-the-art LLMs change quickly. Although we do assess the most recent models from two major providers in this paper at the time of data collection, the results presented here should be treated as a time-limited benchmark, rather than a stable ranking of model performance.

## Conclusion

Our findings indicate that state-of-the-art LLMs can achieve relatively high one-shot accuracy when extracting diverse metascientific variables from psychological research papers, offering substantial gains in speed and scalability. While not yet a substitute for human judgment, their integration into hybrid workflows can streamline meta-research on an unprecedented scale. As models and prompting strategies continue to evolve, LLM-assisted pipelines have the potential to become fundamental to the practice of meta-research itself. By providing a reproducible, open framework for automated data extraction—built on API access, structured JSON outputs, and modular and transparent evaluation—our paper aims to offer both the tools and evidence needed to accelerate effective LLM–human integration in solving meta-research questions.

## Data Availability

The complete prompts and datasets are available in the OSF repository associated with this project: https://osf.io/eqt78/.

## Footnotes

1 The success rates reported in this section differ slightly from the proportions of correct answers provided in Table 1 because the confidence analysis excludes the responses coded as “check” by the models.

## Code availability

The code used for data extraction and analysis, as well as the complete prompts and datasets, is available in the OSF repository associated with this project: https://osf.io/eqt78/
